# Neurocognitive Dysfunction After Treatment for Pediatric Brain Tumors: Subtype-Specific Findings and Proposal for Brain Network-Informed Evaluations

**DOI:** 10.1007/s12264-023-01096-9

**Published:** 2023-08-24

**Authors:** Charlotte Sleurs, Paul Fletcher, Conor Mallucci, Shivaram Avula, Thankamma Ajithkumar

**Affiliations:** 1 Department of Cognitive Neuropsychology, Tilburg University, 5037 AB Tilburg, The Netherlands; 2 Department of Oncology, KU Leuven, 3000 Leuven, Belgium; 3 Department of Psychiatry, University of Cambridge, Addenbrookes Hospital, Cambridge, CB2 0QQ UK; 4 Wellcome Trust MRC Institute of Metabolic Science, University of Cambridge, Cambridge Biomedical Campus, Cambridge, CB2 0QQ UK; 5 Department of Neurosurgery, Alder Hey Children’s NHS Foundation Trust, Liverpool, L14 5AB UK; 6 Department of Radiology, Alder Hey Children’s NHS Foundation Trust, Liverpool, L14 5AB UK; 7 Department of Oncology, Cambridge University Hospital NHS Trust, Cambridge, CB2 0QQ UK

**Keywords:** Connectome, Pediatric brain tumor, Neuroimaging, Neurocognition, Neuropsychological assessment

## Abstract

The increasing number of long-term survivors of pediatric brain tumors requires us to incorporate the most recent knowledge derived from cognitive neuroscience into their oncological treatment. As the lesion itself, as well as each treatment, can cause specific neural damage, the long-term neurocognitive outcomes are highly complex and challenging to assess. The number of neurocognitive studies in this population grows exponentially worldwide, motivating modern neuroscience to provide guidance in follow-up before, during and after treatment. In this review, we provide an overview of structural and functional brain connectomes and their role in the neuropsychological outcomes of specific brain tumor types. Based on this information, we propose a theoretical neuroscientific framework to apply appropriate neuropsychological and imaging follow-up for future clinical care and rehabilitation trials.

## Introduction

Given that the survival rates of pediatric brain tumor patients have increased over time, the quality of daily life increasingly requires attention. As the lesion itself, as well as each treatment constituent can induce neurotoxicity [1], these survivors are at risk of long-term educational or work-related difficulties. Problems in attention, school results, and social functioning can already be observed before or shortly after diagnosis. In addition to these initial symptoms, treatment can lead to additional neural damage. First, neurosurgery causes direct brain tissue damage with related degenerative cascade effects. Since functional brain imaging or direct electrical stimulation during awake surgery is implemented less often in pediatrics, sparing of eloquent areas is highly challenging in this population. Second, cranial radiation can additionally lead to a decline in IQ, with younger patients being more at risk [2]. Even though more precision radiotherapy (RT) techniques, including proton beam irradiation (PBT), yield less scatter radiation and theoretically could be beneficial for cognitive sparing [3], IQ scores and attention [4] can still decline in younger irradiated patients [5]. The beneficial neurological effects of replacing RT with high-dose chemotherapy (CT) in younger patients are still to be elucidated [6]. In addition, how these treatments interact with the developing brain network, or the so-called connectome, needs to be investigated further. In the case of pediatric brain tumors, connectome reorganization primarily depends on the location, size, and histological type of the primary tumor. To understand the functional impact of neural damage on specific brain areas, we provide an overview of the structural and functional brain connectomes involved in cognitive outcomes, which can be translated into the findings of long-term cognitive outcomes for each pediatric brain tumor type. In addition, we propose a new scheme for brain network-informed neurocognitive evaluations and potential rehabilitation strategies in the future.

## Developing Neural Networks with a Neurocognitive Role

**Fig. 1:**
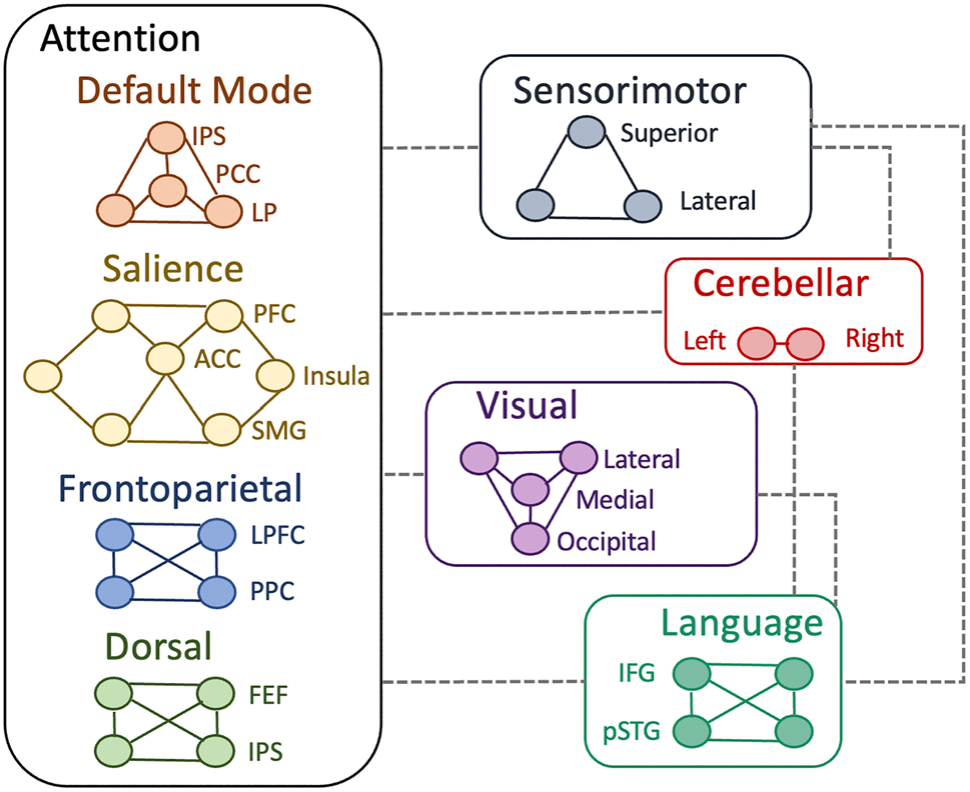
Functional brain areas showing coherent activity associated with certain cognitive or functional tasks. ACC, anterior cingulate cortex; FEF, frontal eye fields; IFG, inferior frontal gyrus (Broca’s area); IPS, intraparietal sulcus; LP, lateral parietal; LPFC, lateral prefrontal cortex; PCC, posterior cingulate cortex; PFC, prefrontal cortex; PPC, posterior parietal cortex; pSTG, posterior superior temporal gyrus (Wernicke’s area); SMG, supramarginal gyrus; Superior, superior section of the S1/M1 areas; Lateral, lateral section of the S1/M1 areas.

**Fig. 2:**
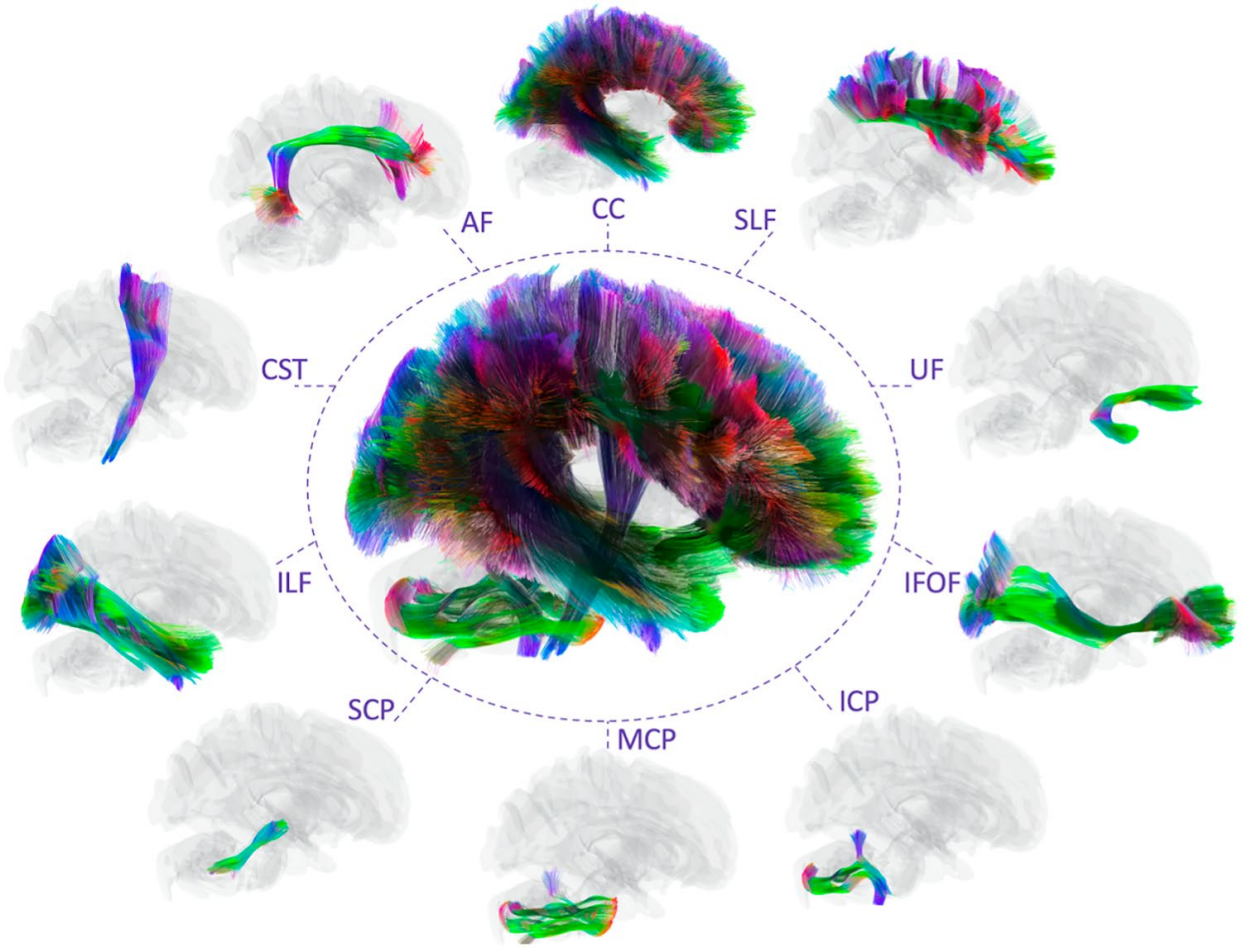
Single delineated white matter tracts. The most well-acknowledged single white matter tracts, being part of the whole-brain tractogram, are depicted in the center of the figure. Example tracts were derived from the HCP tractography atlas and created using DSI studio [119]. AF, Arcuate Fasciculus, CC, Corpus Callosum, CST, Corticospinal Tract, ICP, Inferior Cerebellar Peduncle, IFOF, Inferior Fronto-Occipital Fasciculus ILF, Inferior Longitudinal Fasciculus, MCP, Middle Cerebellar Peduncle, SCP, Superior Cerebellar Peduncle, SLF, Superior Longitudinal Fasciculus, UC, Uncinate Fasciculus.

Multiple functional brain subnetworks are involved in daily life neurocognitive functioning (Fig. 1). There is growing evidence that such networks emerge in the first postnatal year, with increasing network integration as well as segregation, referring to the functional specialization of brain subnetworks [7]. The sensorimotor and visual networks, responsible for sensorimotor perception and the processing of visual information, develop first [8]. Networks associated with language, attention, and executive function develop later on. Although the specialized roles of attention-related networks have yet to be elucidated, a useful heuristic has been to envisage the salience, frontoparietal, and dorsal attentional networks as related to external processing while the default mode network is primarily related to internal processing and representations of one's self and past. All of these networks are well-integrated to produce complex human behavior and cognitive outcomes related to attention, learning, memory, and executive function. They have been defined mainly using functional neuroimaging, which is based on the activity of single regions. The correlated activity of two different regions is often referred to as "functional connectivity". While functional connectivity does not necessarily always imply a structural connection (in the form of white matter tracts or fiber bundles), there is evidence that the functionally-defined networks are underpinned by white matter connections (Fig. 2). Crucially, though, there is more to the network than the inter-regional tracts. Connectivity rather seems to obey a "small-world" principle, whereby some regions act as “railway stations” or so-called hubs, showing both local connections to neighboring regions and more distant connections to other hubs. With a growing understanding of these principles and the particular critical structural and functional hubs, it is becoming increasingly realistic to link impaired cognitive function in children with brain tumors, to the structural disturbances caused by these tumors (and their treatment). In turn, this promises a greater capacity to predict longer-term outcomes and, potentially, to implement targeted neurorehabilitation interventions exploiting the great potential for neuroplasticity.

## Modern Imaging in Assessing Neural Networks in Pediatrics

**Fig. 3:**
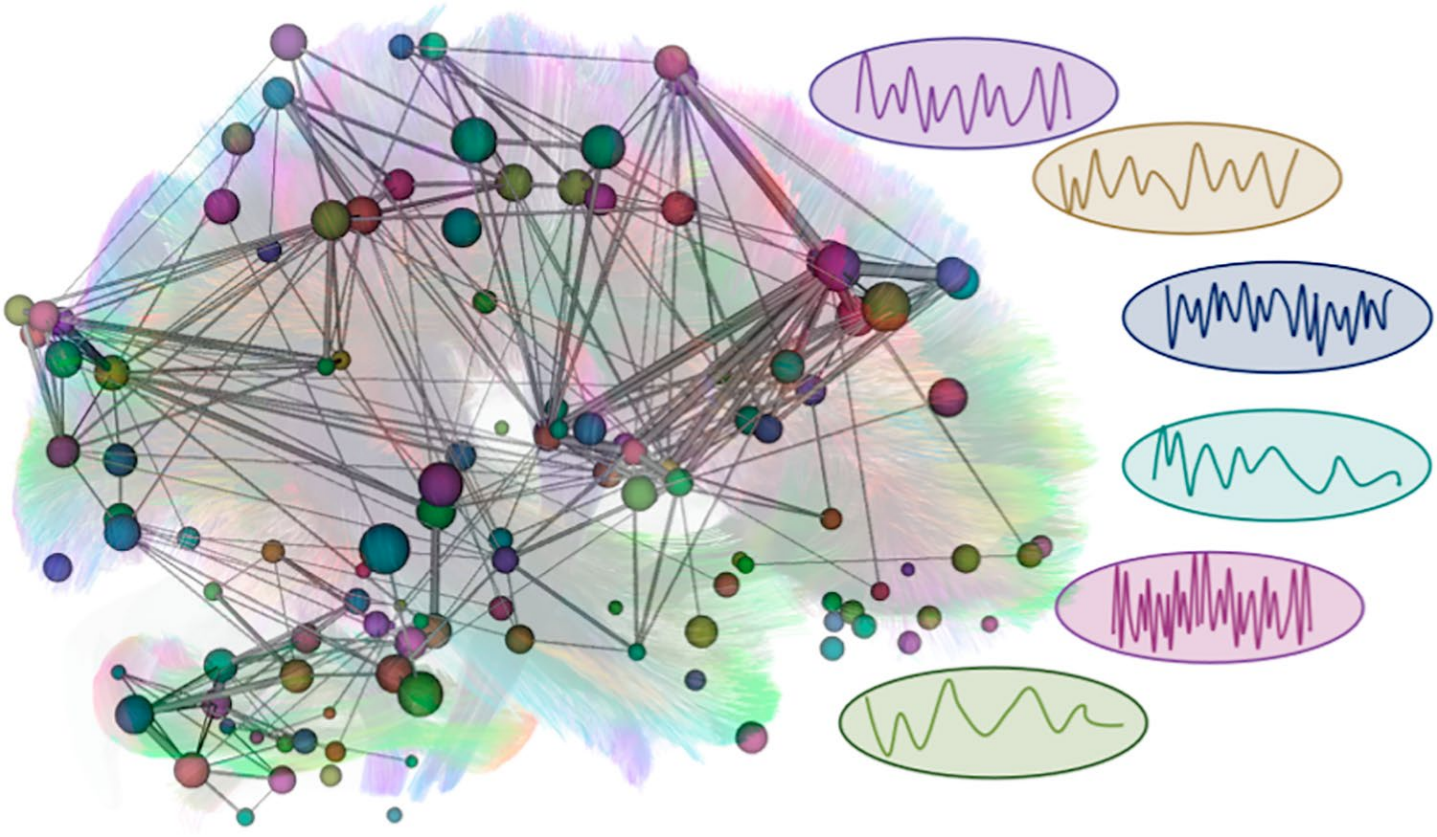
Connectome graph demonstrating the complexity of the existing functional and structural brain networks. A connectome consists of nodes (i.e. regions, depicted as circles) and edges (i.e. representation of a connectivity measure between two regions, depicted as grey lines). In this graph, both the whole-brain tractogram (i.e. estimated streamlines) and regional activity patterns are depicted, which determine connectivity measures of structural and functional connectivity, respectively. For the streamlines in the background, blue indicates tracts along the axial axis (superior-inferior), green indicates tracts along the sagittal axis (anterior-posterior), and red indicates tracts along the coronal axis (left-right). Different nodes are indicated in different colors, presenting different activity patterns. The colored mini-graphs show average activity patterns for different nodes. Based on such a connectome, graph theoretical metrics can be calculated to determine the brain topology. This figure was constructed based on the Human Connectome Project tractography atlas Yeh *et al*. [119], which was complemented with an illustrative functional connectome, using DSI studio. For both the tract atlas and the functional connectome, cortical parcellation was applied according to the AAL-atlas.

**Fig. 4:**
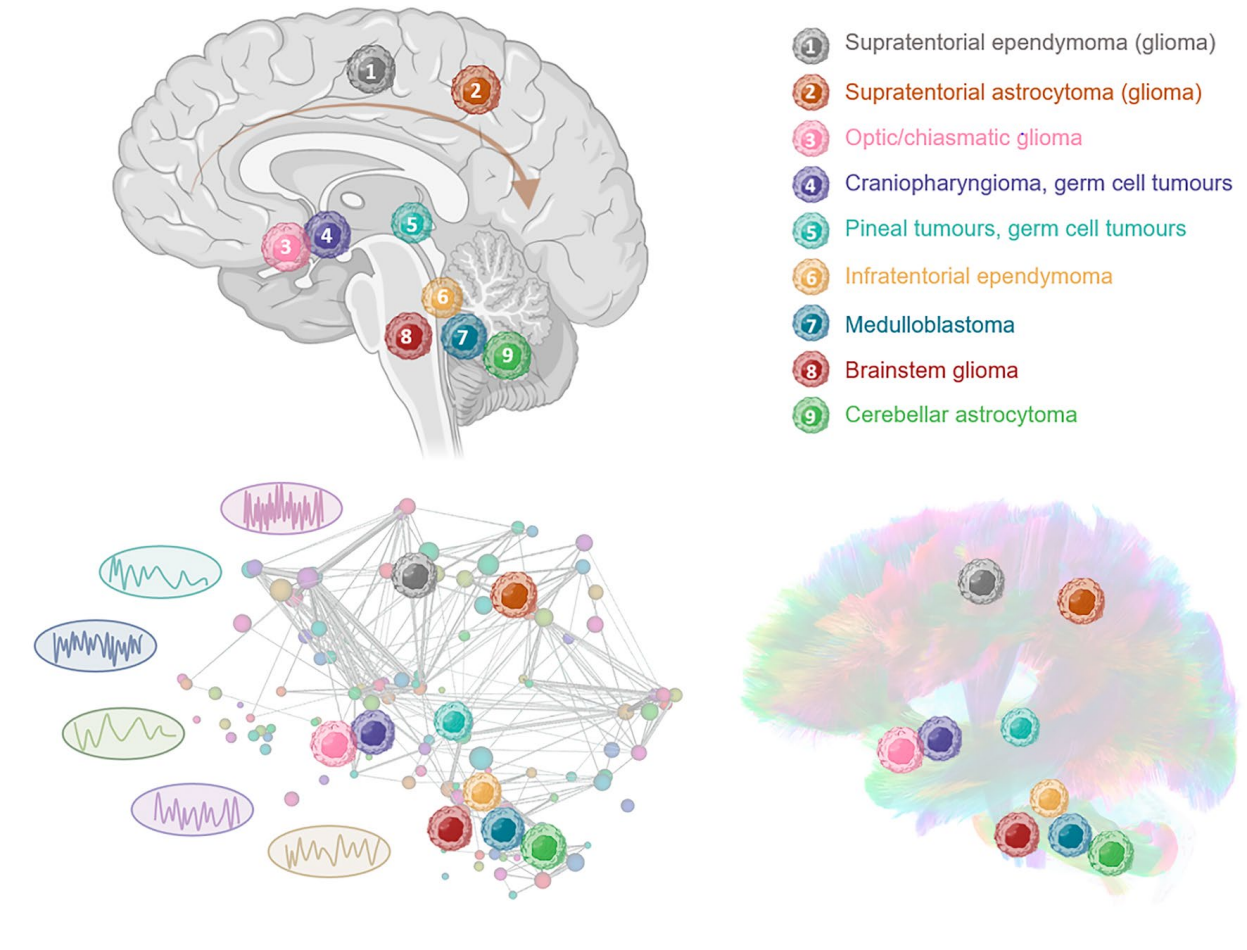
Histology-specific pediatric brain tumor locations and their position in the developing connectome. In the upper figure, the different types of pediatric brain tumors are demonstrated. On the left panel below, these locations of brain tumors are mapped on the whole-brain functional connectome, where different signaling of different regions is demonstrated. On the right panel below, the tumor types are mapped on the whole-brain tractogram, showing which tracts are involved for each subtype.

Regarding conventional neuroradiological approaches, standard anatomical imaging includes contrast-enhanced imaging to detect primary tumors or metastases. However, these imaging techniques do not allow us to estimate brain networks or behavioral outcomes. Hence, to improve the sparing of daily life functional outcomes, we need to move towards the implementation of functional assessments and more advanced imaging techniques. Nowadays, advanced neuroimaging allows us to estimate not only the local micro- and macrostructure of the brain (e.g. cortical thickness, tissue density, gyrification, sulcification) but also estimate the topology of the structural networks and functional coherence or time-dependency (Fig. 3). More specifically, diffusion-weighted MRI allows us to estimate tractograms and consequently structural connectomes. On the other hand, resting-state functional MRI (rs-fMRI) provides an estimate of simultaneous or dynamic oxygen supply, resulting in estimations of functional connectomes. Connectomics is increasingly receiving attention, as cognitive outcomes are not considered to be explained as one-region-specific anymore, but rather as being network-dependent, with the possible importance of hub areas [9]. Connectomics research based on tractography and rs-fMRI can assist in identifying the involved hub areas. In the following sections, specific tumors and associated cognitive outcomes are discussed, with a specific translation to their involved hub areas (Table 1). Histology-specific tumor locations are depicted in Figure 4.

**Table 1:** Overview of hub areas, involved networks, and applicable test assessments for specific risk groups

Cognitive domain	Tumor type & treatment	Eloquent brain areas as potential hubs*	Core functional network	Core structural tracts/network	Example Test assessments
Attention & processing speed	Frontal tumors	Prefrontal Parietal	DAN DMN FPN SAN	Cingulum IFOF SLF	Computerized tasks, CPT, Sustained attention Test of Everyday Attention for Children Wechsler Coding / Symbol search NEPSY Auditory attention
Executive functioning	Frontal/Infratentorial tumors (CMS)	Frontal Motor cortices Cerebellum	DAN FPN SAN Sensorimotor Cerebellar	CST IFOF SLF	Behavioral Assessment of the Dysexecutive Syndrome for Children Delis-Kaplan Executive Function System Wisconsin Card Sorting Test NEPSY Animal sorting
Working memory	Suprasellar tumors (e.g. Craniopharyngioma/ Pituitary/Optic pathway/hypothalamic gliomas)	Prefrontal Medial temporal (including hippocampus)	DMN	Cingulum IFOF, SLF	Wechsler Digit Span backward
Memory	Frontal tumors/ Suprasellar tumors	Prefrontal Medial temporal (including hippocampus)	DMN	Cingulum ILF (visual) UF	Wechsler Digit Span forward Kaufman ABC, Word list learning (e.g. CVLT-C) Wechsler Memory Scales, Stories Dot Locations, Faces
Visuomotor skills	Parieto-occipital tumors/ Infratentorial tumors	Parietal & occipital Motor cortices Cerebellum	Visual Sensorimotor Cerebellar	CST Cerebellar peduncles ILF (visual) SLF (motor)	(Purdue) Pegboard Beery Test of Visual‐Motor Integration Wide Range Assessment of Visual Motor Ability Drawing Test NEPSY Design Copy Wechsler block design
Language	Frontal tumors (speech)/ Temporal tumors (auditory)/ Infratentorial tumors (CMS)	Inferior frontal gyrus (Broca) Superior temporal gyrus (Wernicke)	Language	AF SLF (III)	Language production (object naming) Language perception (auditory) processing, e.g. NEPSY Comprehension, Repetition, Word Generation
Visual perception	Parieto-occipital tumours	Parietal & occipital	Visual	ILF, IFOF SLF (II right) Optic tracts	Visuospatial tasks (location discrimination, mental rotation) Object recognition/naming
Overall developmental stage/ Intelligence/ Fluid reasoning **	Aggressive/larger tumors Intensive treatments (high-dose chemo- and/or radiotherapy, e.g. medulloblastomas)	Whole-brain network topology, efficiency, small-worldness	Whole-brain network topology: all functional units/nodes	Whole-brain network topology: complete tractogram	Development scales/ Intelligence tests (Bayley, Woodcock Johnson, Wechsler, Stanford‐Binet) Matrices reasoning

AF, Arcuate Fasciculus; CMS, Cerebellar Mutism Syndrome; CPT, Continuous Performance Test; CST, Corticospinal Tract; CVLT-C, California Verbal Learning Test for Children; DAN, Dorsal Attention Network; DMN, Default Mode Network; FPN, Frontoparietal Network; IFOF, Inferior Fronto-Occipital Fasciculus; ILF, Inferior Longitudinal Fasciculus; SAN, Salience Network; SLF, Superior Longitudinal Fasciculus; UC, Uncinate Fasciculus.

*The mathematical definition of a “hub” depends on which graph metric is used to estimate the centrality of a region (i.e. node), including nodal degree, nodal efficiency, or betweenness centrality. In the context of neuropsychological outcomes reported in this table, the assumed potential “hubs” are defined based on the eloquent cortical brain areas, of which it is assumed that when impacted, the functional outcome is certainly affected (either directly or indirectly *via* diaschisis).

**Global neurocognitive function, which is mainly affected in case of large neurological impact or toxicity. Hence, such scales are most often associated with whole-brain graph metrics rather than specific eloquent brain areas (e.g. small worldness, global and local efficiency).

## Site-and Tumor-specific Neurocognitive Dysfunction and Imaging Findings

### Infratentorial Tumors

The infratentorial area is the most common location of pediatric brain tumors, with pilocytic astrocytoma (PiA), medulloblastoma (MB), and ependymoma occurring most frequently. PiA is usually treated with surgery only, while treatment of ependymomas and MB can include radiation. Ependymomas are treated with focal treatments (surgery and RT), whereas craniospinal RT is the standard of care for children aged three years or more with MB. These tumors located in or neighboring the cerebellum can infiltrate and induce pressure in this region, affecting mainly motor functions (Figure 1 and Table 1). In this regard, the most well-known acute neurological complication is the so-called cerebellar mutism syndrome (CMS), which is characterized by short-term mutism, reduced speech, hypotonia, and oropharyngeal dysfunction/dysphagia shortly after cerebellar surgery. This often co-occurs with the cerebellar cognitive affective syndrome, including cognitive regulation of affect symptoms as well [10, 11]. These syndromes occur when surgery affects the efferent cerebellar tracts (specifically the dento-thalamo-cortical tracts [10]) but they are also associated with altered supratentorial perfusion [12]. In addition to surgery, cranial RT and systemic CT can also amplify cognitive decline [13].

While PiA presenting as cystic cerebellar lesions are associated with a low risk of neurocognitive dysfunction, an intrinsic lesion or exophytic brainstem or cerebellar peduncle lesion can still cause significant sequelae, similar to more infiltrative tumors. Such symptoms range from difficulties in executive functioning to lower attention and processing speed problems due to front-cerebellar connectivity [14]. Also, patients’ spatial orientation can be affected after surgery [15] as they can suffer from ataxia. Still, the fine motor skills of patients with PiA are better at the group level than patients with an MB [16].

Neuroimaging studies have indicated that the amount of brain tissue resected during surgery is associated with verbal or working memory outcomes [17], which could be associated with secondary demyelination along cerebellar-cortical tracts [18]. Regarding functional brain changes, decreased activity [19] and decreased *N*-acetylaspartate metabolite levels [20] in cerebellar areas could explain the encountered behavioral outcomes.

Literature is lacking on neurocognitive effects in infratentorial ependymoma, probably due to small sample sizes. Still, sequelae can be expected, given the possible involvement of the cranial nerves and the brainstem.

Craniospinal RT in MBs is known to increase the risk of neurocognitive decline, with the most evidence for decreases in intelligence throughout their development [21], with a loss of ~ 2.5 estimated IQ points per year [22]. Early findings of decreases in IQ and executive scores (i.e. within 1 year) can be associated with acute CMS [23], while in the longer run, the negative effects of younger age at treatment and treatment intensity become increasingly apparent [24]. Studies have shown that a higher RT dose to the temporal lobe and hippocampus is associated with lower IQ scale scores [25]. Although replacing RT with high-dose chemotherapy might result in fewer cognitive problems [26], certainly in infants, high-dose chemotherapy is still associated with declines in cognitive scores [27] and with leukomalacia [28] as well.

Besides intellectual decline, executive functioning [29] and fine motor skills [16] are also more severely affected in MB patients than PiA patients, as their craniospinal RT plan involves more brain areas, of which some are eloquent.

Not surprisingly, in neuroimaging studies, atrophic processes in MB can occur across the entire brain. Cerebellar atrophy in MB patients is specifically associated with information processing, attention [30], executive functioning [31], and working memory [19]. These outcomes can also be explained by decreased cerebellar-cerebral connectivity [29, 32]. In addition, hippocampal atrophy [33] can occur post-treatment, and this is associated with decreased memory performance. Other imaging features that are associated with the intelligence outcomes of MB patients consist of white matter volume [34], white matter lesions (e.g. 16% after 8 months [35]), and microstructural alterations [18], of which each can be affected by systemic treatment and irradiation. It is hypothesized that RT can induce accelerated cerebrovascular damage in long-term survivors of pediatric brain tumors [36]. The strongest effects of neural changes are found in the case of higher RT doses [37] and intrathecal methotrexate [28], mostly when treated at a younger age [38]. Also, hydrocephalus [39] and ventricular shunt [38], female gender, and seizures [40] are additional risk factors for normal brain tissue development.

In comparison to PiA, MB patients show decreased cerebral blood flow [18], more evident leukoencephalopathy [30], and less cortical thinning throughout development [41].

### Supratentorial Tumors

Supratentorial tumors have different histological subtypes and can be located either centrally (pituitary/pineal gland, basal ganglia) or in the lobe-specific supratentorial cortices. For each supratentorial brain area involved, other functional outcomes can arise (Figure 1 and Table 1). In the following section, we summarize the literature for each specific tumor.

First, germ cell tumors are located at suprasellar, pineal, or basal ganglia sites. Although the IQ scores can be stable and within the normal range throughout treatment [42, 43], the scores can be somewhat lower than the norm [43]. More specifically, if the tumor involves the basal ganglia, a lower IQ has been found compared to the pineal or suprasellar regions [43, 44]. This can partly be explained by microstructural changes in the white matter [45]. Furthermore, in the case of oculomotor or visual problems, the visuospatial and fine motor skills can be affected [42], which can affect visual memory, processing speed [46], and the performance scale of IQ assessments [47]. Such symptoms are more present at baseline when the mass is located in the pineal than the suprasellar area [42], while the cognitive decline is stronger in the latter subgroup [46]. In addition, RT of these tumors can potentially lead to cavernous malformations, which can affect brain development as well [48].

Craniopharyngiomas and pituitary adenomas are specifically located in the suprasellar area, which is not only close to the hypothalamus but also the hippocampal area. They are generally treated with surgery, with or without focal RT. These tumors are generally reported to have more significant morbidities compared with other suprasellar tumors due to the inherent invasive growth pattern (involving the hypothalamus, for example), which leads to an interruption in neuroendocrine networks. Long-term sequelae include cognitive deterioration, socio-emotional symptoms, sleep dysfunction, and neuroendocrine problems [49]. Furthermore, due to the location close to the hippocampus (Table 1), episodic memory problems are most often reported in craniopharyngioma [50]. Acute post-surgical difficulties can arise in encoding and memory recall, both for visual [51] and verbal information [52]. Such problems are most prevalent in craniopharyngioma involving the hypothalamus [53] (being proximal to the hippocampal areas and more invasive than pituitary tumors). These problems appear worse in the case of hydrocephalus, shunt insertion [54], or growth into the third ventricle [55]. Due to disruptions of thalamocortical and supratentorial tracts (Table 1 and Figure 2), but also to functional and endocrine changes [56], executive functioning can be altered as well as processing speed [57] or impulsivity [58] and working memory problems [59] in particular. An important underlying mechanism could be the hypothalamic-hippocampal circuitry involvement in impulsivity [60], although such deficits are less frequently reported than memory.

Besides surgery, cranial RT can exacerbate cognitive decline, with the most evidence for memory problems after higher temporal lobe RT doses [61], but also a lower IQ when RT doses exceed > 30–45 Gy in the supratentorial region (specifically in left hippocampal/temporal areas) [62].

Based on imaging findings, memory recall is associated with the functional coupling between the medial prefrontal cortex and the thalamus [50], the grey matter volume of the posterior cingulate cortex [63], and treatment-related microstructural changes in the cingulum [64]. Functional imaging has also demonstrated higher amygdala reactivity when emotional faces are presented to craniopharyngioma patients [65], and this is altered after intranasal oxytocin administration [66].

Pituitary adenomas can be functioning (producing excess hormone) or non-functioning. Given the heterogeneity in endocrine sequelae of these tumors, heterogeneous findings exist regarding their neuropsychological profile. Patients with non-functioning adenomas (NFAs) show decreased verbal memory and processing speed compared to the norm before surgery [67]. These findings can be attributed to locations close to the hippocampus and crossing tracts. Such scores remain stable after surgery, also in the case of suprasellar extension [67, 68]. Postsurgical hypothyroidism can lead to further deterioration [67] due to increased fatigue, hyponatremia, and seizures. Furthermore, decreased cognitive scores can still be detected in 41% of long-term survivors, years after treatment [69], while their intelligence assessments remain within the normal range [70]. In the case of macroadenomas, NFAs can grow upward, compressing the optic chiasma and resulting in vision loss (blurry vision). Limited research on specific cognitive outcomes has been performed in these patients. In patients receiving additional RT (because of a tumor remnant or regrowth), working memory and verbal memory can either decline further [71] or stabilize years after treatment [69]. Gamma-knife radiosurgery is specifically not associated with decreased performance in this population [72].

Patients with functioning pituitary adenomas (FPAs), express more cognitive problems before surgery compared to NFA patients [73]. These patients suffer from hyperpituitarism, of which the most well-known conditions are prolactinomas, acromegaly, and Cushing’s disease. This pre-treatment hormonal overproduction appears to be more important for cognitive outcomes than tumor size. More specifically, negative correlations have been found between adrenocorticotropic hormone (ACTH) levels and arithmetic scores [74], prolactin levels, and memory scores [75], and positive correlations between thyroxine and processing speed, working memory [76], and visual recall [74]. In other words, the relationships between endocrine fluctuations and cognitive performance are hormone-specific in FPA patients. Endocrine changes can lead to difficulties in concentration, learning, planning, and complex attention [77], in addition to problems in memory recall [70], while overall IQ scores are stable. Furthermore, long-term hypercortisolism (i.e. endogenous cortisol) as well as cortisol replacement (e.g. exogenous cortisol) can potentially lead to decreased memory performance [78].

Grey matter volume loss has also been noted in patients with pituitary adenomas. More specifically, Cushing’s disease in FPA is associated with decreased grey matter volume in the anterior cingulate cortex [79] and cerebellum [80], which can be associated with the activity of the disease (i.e. ACTH and serum cortisol levels) [81]. Although NFA patients can demonstrate leukoencephalopathy after treatment (certainly in cases with high blood pressure), such lesions are not associated with cognitive outcomes [82]. With regard to functional imaging, decreased working and verbal memory scores are associated with altered perfusion in the temporal areas [71]. Furthermore, electroencephalographic studies have suggested alterations in conflict monitoring [83].

Finally, visual impairment can occur in case of altered connectivity between visual and higher-order areas [84]. Patients with supratentorial tumors who are even more at risk for visual disturbance have hypothalamic gliomas involving the optic pathway and chiasma [85]. However, this population remains to be underinvestigated to date.

Finally, for lobe-specific supratentorial ependymomas/astrocytomas in pediatrics, no studies have focused on the cognitive outcomes of these specific histological subtypes. Depending on the tumor locations, specific subnetworks can be affected, and result in certain behavioral sequelae in daily life (Table 1).

## Challenges of Assessing Neurocognitive Dysfunction After Treatment for Pediatric Brain Tumors

Even though the amount of literature on cognition in pediatric brain tumors is growing exponentially, concerns exist regarding current approaches. More specifically, the existing literature is highly heterogeneous due to the variability of this population, with many confounding factors. These factors include histological tumor types, multimodal treatments with differential surgical approaches and therapeutic doses, wide age ranges, hemisphere dominance, and institution-specific findings due to the lack of multicenter studies.

Not only irradiation and younger age are significant risk factors [5], but also histological tumor type, location, and size [31], relapse [86], epilepsy, endocrine problems [74], hydrocephalus [39] or ventricular shunt [38], cerebellar mutism/cognitive-affective syndrome [23], anesthesia exposure [87], female gender, and seizures [88] can play a major role in daily life neurobehavioral outcomes. In addition, hearing loss, which can be induced by chemotherapy (e.g. platinum-based agents) [89] and RT, can negatively affect neurocognitive outcomes [90]. Hence, intensity-modulated RT [91] and PBT [92] can be beneficial for cognitive measures by sparing healthy brain tissue and decreasing ototoxicity. In addition, multiple single nucleotide polymorphisms have been associated with neurocognitive outcomes [93]. In pediatric brain tumors specifically, most studies evidenced the catechol-O-methyltransferase (COMT) gene involved in neurotransmission [94] and antioxidant enzyme genes (e.g. glutathione S-transferase Theta 1 (GSTT1) and glutathione S-transferase Mu 1 (GSTM1) [95] to be possibly involved. The question for future research remains how these risk factors in addition to a brain tumor interact with the development of the human brain connectome. In this review, we provide a framework for future studies (see below).

## Appraisal of Clinical Tools for Assessing Pediatric Neurocognitive Function

Given the heterogeneity in assessment tools, guidelines have been constructed to homogenize test materials and timing. In Europe, the *SIOPe Quality of Life Group* has proposed that patients below the age of 5 be screened [96] using at least measurements of the developmental quotient (<4 years), and (>4 years) receptive and expressive language, matrices, processing speed, number recall, fine motor, visual motor, semantic memory, and long-term memory. Above the age of 5 years, tests can additionally include the fine motor pegboard and sustained attention and processing speed [97]. These cognitive screenings are suggested to take place at baseline (within the first 6 weeks after diagnosis), 2 years, 5 years post-diagnosis, and 18 years old. This procedure was mainly proposed to standardize assessments across studies, and thus to apply in trials across the pediatric brain population. By contrast, some clinicians might prefer to define time points at the individual level, e.g. to look at specific treatment effects.

While this is a proposed core battery of tests to assess neurocognitive dysfunction, these measures should additionally be associated with neuroimaging parameters, including morphological features of the tumors and non-lesion brain volumes, to improve our knowledge of which areas are crucial to spare from toxicity in the future. This can be a challenge in routine clinical practice. Hence, automated methods of image segmentation and parcellation need to be developed, e.g. for the segmentation of tumoral tissues, as well as regions to be spared (i.e. “organs at risk”). Even more, such optimizations will need to take anatomical deformations into account, using normative templates or healthy tissue as a reference. In order to take the involved networks into account, the selection of cognitive tests could be informed by the tumor-, treatment- and location-based information (Table 1). More specifically, initial “key” tests can be selected and prioritized based on the functional hub (i.e. region) that is involved. In addition, we propose to expand the assessment measuring related or “connected” functions as well (e.g. verbal comprehension as well as speech).

For instance, given that more eloquent areas can be affected by craniospinal or whole-brain RT compared to focal treatment, patients with infratentorial tumors should receive more elaborate testing post-CSI (e.g. IQ, executive function, and visuomotor skills). Similarly, supratentorial tumors close to the medial temporal lobe, such as craniopharyngiomas, can cause more working, verbal, and visual memory problems. Hence, the involved functional hubs should be considered.

## Proposal for a Unified Approach to Measure Neurocognition in Combination with Its Neural Correlates

Regarding functional brain imaging, the acquisition of valid active fMRIs can be challenging in a pediatric population (due to anxiety, excessive movement, boredom, and little understanding of a complicated task). The same limitations count for awake electrical stimulation during neurosurgery as well. Therefore, most of the functional imaging studies outlined above in children applied resting-state fMRI to estimate functional brain networks. However, since functional connectivity in the resting state is a state-dependent correlative measure, it does not provide sufficient information about the causal role of brain regions in functional outcomes, nor about underlying structural connectivity. Furthermore, it cannot be interpreted without taking the state of mind of the individual into account [98]. What complicates the interpretation of resting-state fMRI even more, is when children are too young and sedation is needed; this can affect brain signaling [99]. Future research is therefore needed to determine which functional brain areas apply as hub regions for which behavioral outcomes in healthy children. In addition to these challenges in pediatrics, the definition of a “hub” in brain networks depends on which graph metric is used; this is mostly related to the centrality of a node. These metrics often include nodal degree, nodal efficiency, or betweenness centrality. However, such individual metrics can provide different results on what brain areas are assumed to be the hub regions. Hence, hubness can alternatively be defined based on a combination of graph metrics. Van den Heuvel *et al.* [100] proposed such a combination of nodal metrics (nodal strength, betweenness centrality, clustering coefficient, and characteristic path length), leading to a nodal hub score on a scale of 0–4. More specifically, each nodal metric increased by 1 point if the nodal value was within the 20% highest values. Such an approach could be a more reliable method to estimate hubs within a functional or structural connectome.

In addition, how brain networks reorganize throughout the child’s brain development (and thus the changing microstructural brain environment), up to adulthood, incorporating resting state fMRI and validation against active fMRI is required. Current studies are very limited in acquiring longitudinal data in both pediatric brain tumor patients and controls [32, 101] due to practical challenges. In this context, recent international large-cohort databases have allowed us to use better estimates of normative brain development, including anatomical cortical development as well as network changes [102] and their relationships [103], from the neonatal stage [104] to adulthood [105]. Regarding analyses, it would therefore be advisable to use age- and modality-specific normative templates and parcellations that have been developed for T1-weighted MRI [106], susceptibility mapping [107], perfusion [108], functional networks [109], and diffusion-weighted MRI [110, 111]. Given that functional networks have been generally defined based on the adult population [112], more research on functional pediatric atlases is needed. To handle deformations in the case of pediatric brain tumors, new techniques to artificially correct deformations or lesions are being developed [113] and should be further validated.

**Fig. 5:**
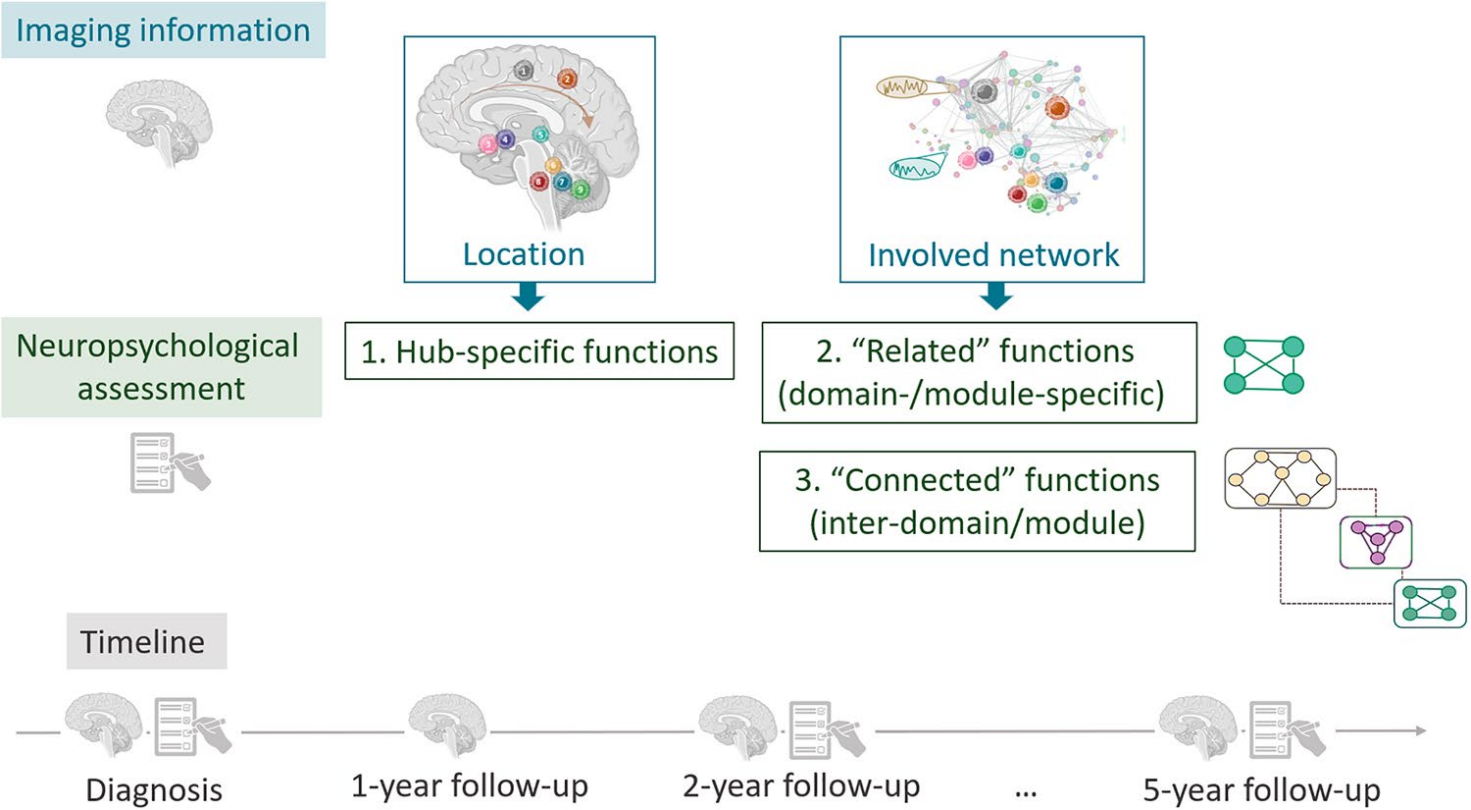
Flowchart for neuroimaging-informed neuropsychological assessment. This figure explains the selection procedure of tests in clinical practice based on tumor location and network information. First, the brain tumor location is used as prior information, to estimate which functional hub is certainly affected. This core network is neuropsychologically assessed in the first place. For example, speech can be assessed in case of damage to Broca’s area. Second, related functions are assessed. For speech, this could be verbal comprehension as an example. Third, functions that can be secondarily affected by changes in the brain network are assessed in addition. For the speech example, this could be auditory sustained attention, which is “connected” to speech function *via* the arcuate fasciculus and superior longitudinal fasciculus.

As neuroimaging findings are scarcer than neurocognitive studies, we recommend that centers obtain uniform prospective imaging data of sufficient quality to perform statistical analyses. This includes high-resolution anatomical scanning (e.g. T1-weighted or T2-weighted MR scanning, < 1 mm isotropic resolution) as well as future imaging studies with sufficiently large samples, including yearly functional and structural connectomics data acquisition [e.g. resting state fMRI and diffusion MRI (including b = 1000)]. Once centers have acquired these data in a standardized way, pre-and post-surgical neural development, as well as RT dosimetry plans, can be investigated more in-depth regarding their neural sequelae and associations with cognitive measurements. Ideally, advanced imaging acquisition (including connectomics data) would take place as close as possible to the time points of the standardized neuropsychological assessments. In other words, this would at least take place at baseline (within the first 6 weeks after diagnosis) and at 2-year follow-up (Fig. 5). Trials can then be performed comparing hub information and functional outcomes in targeted neurocognitive rehabilitation arms and control arms with the standard of care.

Finally, throughout the procedures of neuropsychological and advanced imaging follow-up, it is important to incorporate shared decision-making with parents as much as possible. Although this can be challenging in pediatric oncology [114], examples of prognostic communication have recently been proposed for pediatric patients with neurological conditions [115].

## Current Conclusions Towards Treatment Optimization

Regarding neurosurgery, subtotal resection is intrinsically associated with less brain injury. However, the disease process itself might result in secondary damaging effects, and thus the aggressiveness of the tumor, as well as shared decision-making with parents, are important to keep in mind for such decisions. Awake surgery can additionally be recommended in adolescents with tumors located in eloquent areas. Regarding infratentorial tumors, proximal efferent cerebellar pathways are to be spared in modern neurosurgery as much as possible, without interfering with the superior cerebellar peduncles, to limit the risk of cerebellar mutism/cognitive-affective syndrome. Finally, FPA surgery can improve neurocognitive outcomes when stabilizing the hormonal status.

Second, RT planning can be modified in order to spare the eloquent areas as much as possible, of which the hippocampal area has been proposed as a potentially important hub [62]. However, hub analyses could yield new findings and novel information on tract-based or function-based sparing. In addition, (hyper-)fractionation of RT or PBT [4, 116] could also reduce the cognitive sequelae compared to conventional photon beam therapy.

Finally, interventional studies including pharmacological studies have only recently shown some preliminary positive evidence for metformin [117]. Furthermore, behavioral interventions such as physical exercise can improve neural sparing [118]. In conclusion, efficient sparing of hub areas associated with specific cognitive functions from atrophy or any treatment-induced toxic mechanisms will require large-scale imaging-neurobehavioral research in this population in the near future.
